# Timing of positive blood samples does not differentiate pathogens causing healthcare-associated from community-acquired bloodstream infections in children in England: a linked retrospective cohort study

**DOI:** 10.1017/S0950268814003306

**Published:** 2014-12-08

**Authors:** K. L. HENDERSON, B. MÜLLER-PEBODY, A. WADE, M. SHARLAND, M. MINAJI, A. P. JOHNSON, R GILBERT

**Affiliations:** 1 Department of Healthcare-Associated Infection and Antimicrobial Resistance, Centre for Infectious Disease Surveillance and Control, Public Health England, London, UK; 2 Paediatric Epidemiology and Biostatistics, UCL Institute of Child Health, London, UK; 3 Paediatric Infectious Diseases Unit, St George's Hospital, London, UK

**Keywords:** Bloodstream infections, hospital-acquired (nosocomial) infections, microbiology, paediatrics

## Abstract

Paediatricians recognize that using the time-dependent community-acquired *vs.* hospital-acquired bloodstream infection (BSI) dichotomy to guide empirical treatment no longer distinguishes between causative pathogens due to the emergence of healthcare-associated BSIs. However, paediatric epidemiological evidence of the aetiology of BSIs in relation to hospital admission in England is lacking. For 12 common BSI-causing pathogens in England, timing of laboratory reports of positive paediatric (3 months to 5 years) bacterial blood isolates were linked to in-patient hospital data and plotted in relation to hospital admission. The majority (88·6%) of linked pathogens were isolated <2 days after hospital admission, including pathogens widely regarded as hospital acquired: *Enterococcus* spp. (67·2%) and *Klebsiella* spp. (88·9%). *Neisseria meningitidis, Streptococcus pneumoniae*, group A streptococcus and *Salmonella* spp. were unlikely to cause hospital-acquired BSI. Pathogens commonly associated with hospital-acquired BSI are being isolated <2 days after hospital admission alongside pathogens commonly associated with community-acquired BSI. We confirm that timing of blood samples alone does not differentiate between bacterial pathogens. Additional factors including clinical patient characteristics and healthcare contact should be considered to help predict the causative pathogen and guide empirical antibiotic therapy.

## INTRODUCTION

There is increasing evidence that the traditional categorization of bloodstream infections (BSIs) in children as either community acquired (CA) or hospital acquired (HA) is no longer adequate to guide empirical treatment of sepsis, as patients presenting from the community may have infections that are actually healthcare-associated (HCA) and caused by pathogens acquired in a healthcare setting, rather than the community [1]. Since the 1980s, a 48-h post-admission threshold for CA- *vs.* HA-BSI has commonly been used to guide empirical antibiotic treatment based on the different pathogens circulating in the community *vs.* the hospital environment [2–9]. However, the epidemiology of paediatric BSIs has since evolved due to medical advances, shorter length of hospital stay and the ability to manage complex conditions, including those requiring the use of invasive devices, at home. As a result, children presenting to hospital with systemic infections may have either a CA- or HCA-BSI, with the latter more likely to be caused by pathogens commonly associated with HA-BSI, which require different empirical therapy to CA-BSI [1, 10–12]. Currently, the bacterial aetiology of paediatric BSIs in relation to hospital admission in England is unknown and evidence to guide the formulation of recommendations for treatment of paediatric BSIs is lacking.

A recent systematic review found only two regional studies, from Canada and England, that reported the proportions of paediatric HCA-BSI since 2000 [13]. To address this gap in our understanding of the aetiology and epidemiology of paediatric BSIs in England, we used national data to analyse the timing of positive blood samples from children in relation to their hospital admission for 12 common pathogens causing BSI.

## METHODS

### Linked national laboratory and clinical data

We used probabilistic methods to link retrospective national BSI microbiology surveillance data and hospital administrative data (Hospital Episode Statistics; HES) for children aged 3 months to 5 years admitted to a National Health Service (NHS) hospital or treated by the NHS in a private hospital in England between 1 April 2009 and 31 March 2010. Children aged 3 months to 5 years have the second highest incidence rate of paediatric BSI after neonates [14]. Neonates aged <3 months were excluded to avoid BSIs resulting from vertically transmitted pathogens and HA-BSI acquired by babies in intensive-care units since birth. The microbiology surveillance data were voluntarily submitted to Public Health England's (PHE) database (LabBase2) by laboratories across England. LabBase2 captures data from a substantial proportion of all positive blood specimens (for example, 80% of cases of BSI caused by methicillin-resistant *Staphylococcus aureus* (MRSA) reported via the national mandatory reporting scheme are also reported to LabBase2 [15].

The following patient identifiers were used to link the datasets: NHS number, hospital number, date of birth, sex and postcode. Match-weights for the probabilistic linkage were calculated using the following equation:

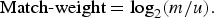

A standard reference dataset of linked LabBase2 and HES data from 2009 to 2010 considered to be true links was used to calculate the match-weights. The frequency of match-pairs that agreed, disagreed or were missing particular identifiers from this reference dataset were summed for each patient identifier (*m* value; matched). The true links match-pairs were then internally cross-matched to create all possible false-match combinations from the standard reference dataset to calculate the frequency counts of match-pairs that agreed, disagreed or were missing values by chance (*u* value; unmatched). The match-weight threshold for inclusion was selected following manual review of ≤100 randomly sampled match-pair observations per match-weight above the value of zero by K.L.H., and the highest match-weight for each laboratory blood specimen report was kept above this threshold. A *χ*^2^ test was used to assess whether the unlinked and linked datasets were significantly different by gender, age and pathogen type to identify any linkage bias.

The objective of the analysis was to assess the association between the bacterial aetiology of BSI and the timing of positive blood specimens taken before and during admission to hospital. Following linkage, BSI reports were selected for inclusion in the analysis where the pathogen was isolated between 5 days before and up to 30 days after hospital admission. Only the first reported positive blood specimen per patient was included in the analysis and we focused on 12 frequently isolated pathogens within this age group: *S. aureus* [split into MRSA and methicillin-susceptible *S. aureus* (MSSA) where a methicillin susceptibility result was reported], non-pyogenic streptococci, *Enterococcus* spp., group B streptococcus (GBS; *Streptococcus agalactiae*), *Streptococcus pneumoniae*, group A streptococcus (GAS; *Streptococcus pyogenes*), *Escherichia coli, Klebsiella* spp., *Enterobacter* spp., *Pseudomonas aeruginosa, Salmonella* spp., and *Neisseria meningitidis*.

It should be noted that the exact time in hours of a blood specimen or the admission to hospital were not reported in either dataset; therefore ‘day’ is the closest approximation and may introduce a day-drift effect. For example two patients may be admitted on the same day, but one at 01:00 hours and other at 23:00 hours, leaving a 22-h difference. Patients may also have gone to an accident and emergency (A&E) department or NHS walk-in centre before being admitted to hospital as an in-patient. Records for A&E visits are held in a separate database that was not consulted for this study; however, we used data on the source of admission to explain positive blood specimens before admission to hospital. Isolates taken ⩾2 days after admission are consistent with the HA-BSI definition using a 48-h cut-off most widely used in the published literature [13].

### Ethics approval

Public Health England has National Information Governance Board for Health and Social Care (NIGB) approval for the collation of surveillance data in accordance with section 251 of the NHS Act 2006.

## RESULTS

### Linkage of national laboratory and clinical data

**Fig. 1.**
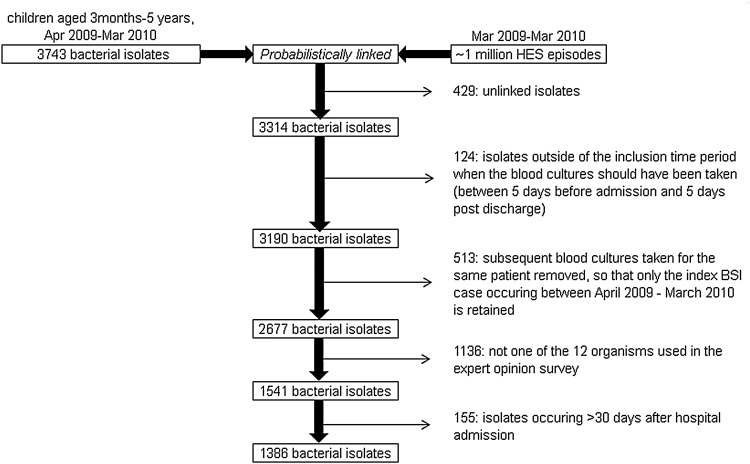
Flow diagram illustrating the inclusion criteria for the linked national laboratory and clinical data. HES, Hospital Episode Statistics

There were 3743 reports of positive blood samples from children aged 3 months to 5 years during the study period, of which 89% (*n* = 3314) linked to HES records (Fig. 1); patient identifier completion was poor in the unlinked records (21% lacked NHS number, 31% lacked postcode). The type of pathogen was significantly different between the linked and unlinked datasets (*χ*^2^ *P* = 0·001). There was a higher proportion of GAS (8·2% *vs*. 3·9%), *N. meningitidis* (7·2% *vs*. 4·7%) and GBS (1·6% *vs*. 0·3%) in the unlinked compared to the linked datasets, and a lower proportion of *S. pneumoniae* (5·6% *vs*. 8·1%) and non-pyogenic streptococci (5·6% *vs*. 8·1%) in the unlinked dataset compared to the linked dataset; age and gender distributions were not significantly different. Of these, 124 isolates were removed if they did not occur between 5 days before and 5 days after hospital admission, and 513 subsequent blood specimens were removed so that only the index BSI case per child remained, leaving a total of 2677 isolates.

A total of 1386 index BSI records for the 12 common pathogens were reported for cases where the pathogen was isolated from blood between 5 days before and 30 days after admission to hospital. Of these, 57% were male and the median age was 1·73 years.

### Characteristics of BSI caused by different pathogens

**Fig. 2.**
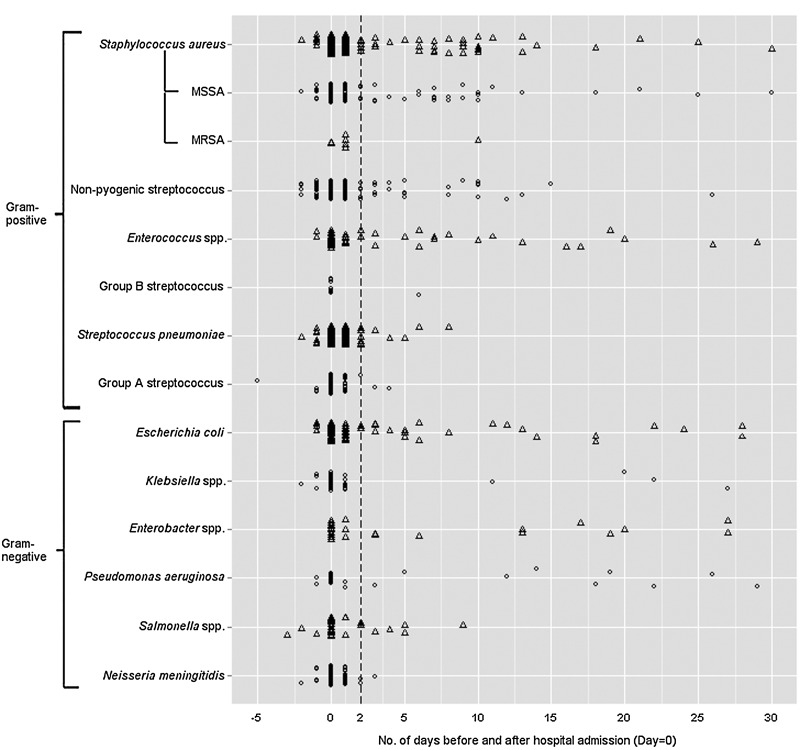
Distribution of the timing of positive blood specimens in relation to hospital admission (day = 0) for the 12 most frequently reported pathogen groups. The dotted line illustrates 2 days threshold after hospital admission.

The number of positive blood samples together with their timing in relation to hospital admission is shown for each pathogen in Figure 2. Seventy (5%) patients had a positive blood specimen 2 days (*n* = 68) or >2 days (*n* = 2) before their admission to hospital. Of the 68 cases, 55 were admitted as emergency cases to hospital from the A&E department, seven were admitted as emergency cases from other sources including the A&E department of other healthcare providers, three were emergency cases from general practitioners (GPs), one case was transferred from another hospital, one case had a planned elective admission and one case was admitted from an outpatient clinic. For the remaining two cases, the case whose blood specimen was taken on day 3 was admitted from the A&E department; the case whose blood specimen was taken 5 days before admission was an emergency GP admission.

Table 1 reports that 88·6% [95% confidence interval (CI) 88·5–88·7] of all 12 pathogen groups (range 60–98%), were isolated <2 days after hospital admission. Grouped by specific pathogens, 67·2% (95% CI 46·6–84·2) of *Enterococcus* spp., 88·9% (95% CI 49·0–100) of *Klebsiella* spp. and 64·0% (95% CI 0–100) of *P. aeruginosa*, and over 90% of all streptococci were isolated <2 days after hospital admission. Ninety-five per cent of *S. pneumoniae*, GAS, *N. meningitidis, Salmonella* spp. and GBS were isolated <2 days after hospital admission. Full details are shown for all pathogens causing BSIs between 5 days before and 30 days after hospital admission (*n* = 2435) in Supplementary Appendix A (online).

**Table 1.** The proportional distribution by time of 12 pathogen groups of 1386 positive bacterial isolates between 5 days before and 30 days after hospital admission in children aged 3 months to 5 years, England

Gram	Pathogen	Total no. reported	Days either side of admission to hospital (day = 0)					
			−5 d to +1 d	(95% CI)	+2 to +5 d	(95% CI)	+6 to +30 d	(95% CI)
Positive	*Staphylococcus aureus*	227	83%	(81–85)	5%	(4–6)	12%	(11–15)
	MSSA	187	85%	(82–88)	5%	(4–7)	10%	(8–13)
	MRSA	7	86%	(0–100)	0%	(0–100)	14%	(0–100)
	Non-pyogenic streptococcus†	331	92%	(91–93)	5%	(4–6)	3%	(3–4)
	*Enterococcus* spp.	61	67%	(47–84)	8%	(0–24)	25%	(10–44)
	Group B streptococcus	8	88%	(0–100)	0%	(0–100)	13%	(0–100)
	*Streptococcus pneumoniae*	253	95%	(94–96)	4%	(3–5)	1%	(1–2)
	Group A streptococcus	123	98%	(94–99)	2%	(1–6)	0%	(0–3)
Negative	*Escherichia coli*	113	80%	(72–95)	8%	(5–14)	12%	(9–19)
	*Klebsiella* spp.	36	89%	(49–100)	0%	(0–27)	11%	(0–50)
	*Enterobacter* spp.	25	60%	(0–100)	8%	(0–76)	32%	(0–100)
	*Pseudomonas aeruginosa*	25	64%	(0–100)	8%	(0–76)	28%	(0–100)
	*Salmonella* spp.	33	79%	(28–100)	18%	(0–67)	3%	(0–40)
	*Neisseria meningitidis*	151	98%	(96–99)	2%	(1–4)	0%	(0–2)
								
	Total*	1386	89%	(89–89)	**5%**	(4–6)	**7%**	(7–7)

CI, Confidence interval.

* The total does not double count the *Staphylococcus aureus* isolates tested for methicillin susceptibility [methicillin-susceptible *S. aureus* (MSSA), methicillin-resistant *S. aureus* (MRSA)].

† Non-pyogenic streptococci includes: *Abiotrophia defectiva, Aerococcus* spp., *S. acidominimus, S. alactolyticus, S. anginosus, S. bovis, S. constellatus, S. dysgalactiae, S. gordonii*, group C streptococcus, group D streptococcus, *S. intermedius, S. milleri* group, *S. mitior, S. mitis, S. mutans, S. oralis, S. parasanguinis, S. salivarius, S. sanguinis, S. sobrinus, S. uberis, S. vestibularis, S. viridans*, other *Streptococcus* spp.

## DISCUSSION

Almost 90% of BSIs for 12 common pathogens in children aged 3 months to 5 years were isolated from samples taken <2 days after hospital admission. As expected, this proportion was even higher for pathogens that are viewed as predominantly community acquired (*S. pneumoniae*, GAS, *Salmonella* spp., *N. meningitidis*). The finding that over two-thirds of pathogens (such as *Enterococcus* spp., *Klebsiella* spp., *P. aeruginosa*), which are usually considered to be hospital acquired, were isolated <2 days after hospital admission, indicates that children presenting with BSIs to hospital are likely to reflect a mixture of CA- and HCA-BSIs [13].

The overall high proportion of BSIs that were detected <2 days after admission is similar to the 81% proportion of CA- and HCA-BSI reported by Laupland *et al.* for children aged 30 days to <18 years in Canada [5]. Evidence is lacking on the timing of positive samples of BSI by pathogen in relation to hospital admission for children in England; an English, one-centre study reported BSIs in children by pathogen but only distinguished between children with and without serious underlying medical conditions rather than CA- or HA-BSI due to a recognition of the changing epidemiology of pathogens causing BSIs and the emergence of HCA infections [16].

The data linkage results, along with our previous review of the paediatric literature and other studies in adult patients argue that the timing of positive blood specimens in relation to hospital admission is insufficient on its own to differentiate CA- from HCA-BSI in order to inform empirical antibiotic therapy [10, 13]. Further analysis of these linked data is needed to develop statistical models to identify patient and clinical characteristics such as age, previous healthcare contact, invasive procedures and underlying chronic conditions that may help to differentiate CA-, HCA- and HA-BSI and to guide empirical treatment.

## Supplementary material

For supplementary material accompanying this paper visit https://doi.org/10.1017/S0950268814003306. click here to view supplementary material
