# Supplementary material for: Timing of positive blood samples does not differentiate pathogens causing healthcare-associated from community-acquired bloodstream infections in children in England: a linked retrospective cohort study
Source: Epidemiol Infect. 2014 Dec 8;143(11):2440–5. doi: 10.1017/S0950268814003306 (PMC4531492; doi:10.1017/S0950268814003306)
Supplement: Supplementary file 1 [file S0950268814003306sup001.doc]

**Appendix A.** The proportional distribution by time of all 2,435 positive bacterial isolates between 5 days before and 30 days after hospital admission in children aged 3 months to 5 years, England

| **Gram** | **Pathogens** | **Total no.** | **Days either side of admission to hospital (day=0)** | | | | | |
| --- | --- | --- | --- | --- | --- | --- | --- | --- |
|  |  | **reported** | **-5d to +1d** | **(95% CI)** | **+2 to +5d** | **(95% CI)** | **+6 to +30d** | **(95% CI)** |
| **Positive** | ***Staphylococcus aureus*** | 227 | 83.3% | (80.9-85.1%) | 4.4% | (3.5-6.0%) | 12.3% | (10.7-14.5%) |
|  | **MSSA** | 187 | 88.2% | (85.3-90.3%) | 4.8% | (3.6-7.0%) | 10.2% | (8.3-12.9%) |
|  | **MRSA** | 7 | 85.7% | (0-100%) | 0.0% | (0-100%) | 14.3% | (0-100%) |
|  | **Non-pyogenic streptococcus** | 331 | 91.8% | (90.8-92.6%) | 4.8% | (4.3-5.7%) | 3.3% | (2.9-4.1%) |
|  | ***Enterococcus* spp.** | 61 | 67.2% | (46.6-84.2%) | 8.2% | (0.6-23.9%) | 24.6% | (9.7-44.3%) |
|  | **Group B streptococcus** | 8 | 87.5% | (0-100%) | 0.0% | (0-100%) | 12.5% | (0-100%) |
|  | ***Streptococcus pneumoniae*** | 253 | 94.9% | (93.5-95.7%) | 4.0% | (3.2-5.2%) | 1.2% | (0.9-2.1%) |
|  | **Group A streptococcus** | 123 | 97.6% | (93.9-98.8%) | 2.4% | (1.1-6.1%) | 0.0% | (0-2.5%) |
|  | **Coagulase-negative staphylococcus** | 635 | 87.7% | (87.3-88.1%) | 5.7% | (5.4-6.0%) | 6.6% | (6.3-7%) |
|  | ***Micrococcus* spp.** | 73 | 94.5% | (83.7-98.9%) | 5.5% | (0.9-16.1%) | 0.0% | (0-6.8%) |
|  | **Diphtheroids** | 59 | 89.8% | (72.2-98.8%) | 5.1% | (0-20.0%) | 5.1% | (0-20%) |
|  | **Other Gram-positive pathogensⱡ** | 53 | 79.2% | (55.1-95.5%) | 9.4% | (0-29.8%) | 11.3% | (0-32.5%) |
|  |  |  |  |  |  |  |  |  |
| **Negative** | ***Escherichia coli*** | 113 | 79.6% | (72.2-85.3%) | 8.0% | (4.7-13.7%) | 12.4% | (8.1-18.9%) |
|  | ***Klebsiella* spp.** | 36 | 88.9% | (49.0-100%) | 0.0% | (0-26.8%) | 11.1% | (0-50.4%) |
|  | ***Enterobacter* spp.** | 25 | 60.0% | (0-100%) | 8.0% | (0-75.6%) | 32.0% | (0-100%) |
|  | ***Pseudomonas aeruginosa*** | 25 | 64.0% | (0-100%) | 8.0% | (0-75.6%) | 28.0% | (0-100%) |
|  | ***Salmonella* spp.** | 33 | 78.8% | (28.3-100%) | 18.2% | (0-67.1%) | 3.0% | (0-40.2%) |
|  | ***Neisseria meningitidis*** | 151 | 98.0% | (95.5-98.8%) | 2.0% | (1.1-4.4%) | 0.0% | (0-1.6%) |
|  | ***Haemophilus influenzae*** | 28 | 85.7% | (23.7-100%) | 7.1% | (0-62.3%) | 7.1% | (0-62.3%) |
|  | ***Moraxella* spp.** | 40 | 95.0% | (65.9-100%) | 2.5% | (0-28.4%) | 2.5% | (0-28.4%) |
|  | ***Pseudomonas* spp.** | 18 | 83.3% | (0-100%) | 11.1% | (0-100%) | 5.6% | (0-100%) |
|  | ***Serratia* spp.** | 2 | 50.0% | (0-100%) | 0.0% | (0-100%) | 50.0% | (0-100%) |
|  | ***Acinetobacter* spp.** | 47 | 91.5% | (66.9-100%) | 6.4% | (0-29.3%) | 2.1% | (0-21.2%) |
|  | **Coliform** | 26 | 88.5% | (20.7-100%) | 3.8% | (0-61.5%) | 7.7% | (0-70.7%) |
|  | **Other Gram-negative pathogens*** | 68 | 85.3% | (70.0-94.7%) | 5.9% | (0.6-18.1%) | 8.8% | (1.9-22.2%) |
|  |  |  |  |  |  |  |  |  |
|  | **Total** | **2,435** | **87.8%** | (87.7-87.8%) | **6.4%** | (6.4-6.5%) | **5.8%** | (5.8-5.8%) |
|  |  |  |  |  |  |  |  |  |
| ⱡ*Actinomyces meyeri, Actinomyces* spp., *Bacillus cereus, Bacillus* spp., *Brevibacterium* spp., *Clostridium beijerinckii, Clostridium clostridiforme, Clostridium septicum, Clostridium tertium, Gemella morbillorum, Lactobacillus* spp., *Lactococcus lactis, Lactococcus* spp., *Leuconostoc* spp., *Rhodococcus* spp., Streptococcus Group F | | | | | | | | |
| **Achromobacter* spp., *Alcaligenes xylosoxidans, Branhamella* spp., *Brevundimonas diminuta, Brevundimonas vesicularis*, *Burkholderia cepacia,* *Campylobacter jejuni, Campylobacter* spp., *Chryseobacterium indologenes*, *Citrobacter freundii*, *Comamonas acidovorans*, *Comamonas testosteroni*, *Eikenella corrodens*, *Escherichia hermannii*, *Fusobacterium nucleatum*, *Haemophilus* spp., *Kingella kingae*, *Morganella morganii*, *Neisseria mucosa*, *Neisseria* spp., *Neisseria Sicca*, *Pantoea* spp., *Pasteurella* spp., *Proteus Mirabilis*, *Sphingomonas paucimobilis*, *Stenotrophomonas maltophilia* | | | | | | | | |
